# Analysis of the effects of depression associated polymorphisms on the activity of the *BICC1* promoter in amygdala neurones

**DOI:** 10.1038/tpj.2015.62

**Published:** 2015-10-06

**Authors:** S Davidson, L Shanley, P Cowie, M Lear, P McGuffin, J P Quinn, P Barrett, A MacKenzie

**Affiliations:** 1 School of Medical Sciences, University of Aberdeen, Foresterhill, Aberdeen, UK; 2 Department of Molecular and Clinical Pharmacology, Institute of Translational Medicine, University of Liverpool, Liverpool, UK; 3 Social, Genetic and Developmental Psychiatry Centre (MRC), Institute of Psychiatry, Psychology and Neuroscience, Denmark Hill, London, UK; 4 The Rowett Institute of Nutrition and Health, Bucksburn, Aberdeen, UK

## Abstract

The Bicaudal C Homolog 1 (*BICC1*) gene, which encodes an RNA binding protein, has been identified by genome wide association studies (GWAS) as a candidate gene associated with major depressive disorder (MDD). We explored the hypothesis that MDD associated single-nucleotide polymorphisms (SNPs) affected the ability of cis-regulatory elements within intron 3 of the *BICC1* gene to modulate the activity of the BICC1 promoter region. We initially established that the *BICC1* promoter drove *BICC1* mRNA expression in amygdala, hippocampus and hypothalamus. Intriguingly, we provide evidence that MDD associated polymorphisms alter the ability of the *BICC1* promoter to respond to PKA signalling within amygdala neurones. Considering the known role of amygdala PKA pathways in fear learning and mood these observations suggest a possible mechanism through which allelic changes in the regulation of the *BICC1* gene in amygdala neurones may contribute to mood disorders. Our findings also suggest a novel direction for the identification of novel drug targets and the design of future personalised therapeutics.

## Introduction

Major depressive disorder (MDD) will affect up to 25% of the adult population at some point in their lives^1^ and represents one of the major causes of morbidity in the Western World^2^ with significant economic consequences.^3^ Although environmental factors such as stress and inflammatory disease have a strong role in the development of MDD,^4, 5^ there is also significant evidence of a genetic contribution to susceptibility although estimates to the scale of the contribution vary.^6^ To identify contributing genetic loci we previously undertook a genome wide association (GWA) analysis of 1346 individuals displaying recurrent depression fulfilling DSM-IV or ICD-10 criteria of at least moderate severity against 1288 unaffected individuals and identified a number of candidate loci that were strongly associated with MDD (rs9416742, *P*=1.3 × 10^−7^ and rs999845, *P*=1.2 × 10^−7^).^7^ Neither of these single-nucleotide polymorphisms (SNPs) occurred within a gene coding region or were found to be in linkage disequilibrium (LD) with any coding SNP. However, both these loci occupied intron 3 of the Bicaudal C Homolog 1 gene (*BICC1*). *BICC1* was originally identified as mouse mutations which truncated the BICC1 protein were shown to result in polycystic kidney disease in mice.^8^ Subsequent analyses showed that inactivation of *BICC1* resulted in Wnt^9^ and PKA^10^ pathway hyperactivation. Biochemical analysis demonstrated that the BICC1 protein contained three K homology domains that are involved in RNA binding and that BICC1 protein is implicated in post-transcriptional regulation of gene expression by antagonising miRNAs.^11^ More recent studies have also implicated the involvement of BICC1 protein in modulating bone density.^12^ Two mRNA isoforms have been reported for *BICC1*; termed the long and the short isoform. Using microarray analysis we detected the expression of the long isoform of *BICC1* in neuronal tissues but were unable to detect significant levels of expression of the short isoform.^7^ Interestingly, the long isoform also displayed significant evidence of differential expression between different brain areas.^7^ Furthermore, MRI brain scan studies of MDD patient's demonstrated that the T allele of rs999845, which was protective against MDD, was associated with greater hippocampal volumes.^13^ Intriguingly, this effect was lost if the patient had been exposed to stressful events during childhood suggesting an epigenetic influence.^13^

The finding of disease associated SNPs within the intron 3 of the *BICC1* gene is consistent with the vast majority of other GWA studies where 93% of disease associated SNPs fall within regions of the genome that do not encode protein.^14^ The discovery that such a high proportion of associated SNPs occur in non-coding regions has hampered efforts to explore the functional consequences of SNPs within the non-coding genome that still remains poorly understood. Clues as to the functional consequences of many disease associated SNPs come from whole genome functional analyses as typified by the ENCODE consortium.^15^ Thus, 73% of disease associated SNPs were detected in regions of the genome that had characteristics of cis-regulatory regions determined using techniques such as FAIRE-seq, DNAseI-seq and ChIP-seq that identify regions of the genome that are less condensed or, which bind transcription factors or specifically modified histones *in vivo*.^14^ These cis-regulatory sequences include promoters, enhancers and silencer elements whose integrity is essential to maintaining appropriate levels of the expression of specific genes, in specific cells and in response to the correct stimuli.^16^ Indeed, the burden of proof now suggests that polymorphic variation within these cis-regulatory elements represents the main reservoir of heritable disease including MDD.^14^

To determine the effects of non-coding SNPs on the tissue specific activity of cis-regulatory elements we have previously used a combination of comparative genomics, reporter assay analysis in transgenic mice and magnetofected primary neurones. In this way we have shown that cis-regulatory regions with strong cell specificity can be identified by virtue of their high degrees of evolutionary conservation.^17, 18, 19, 20, 21^ Furthermore, we have demonstrated that the activity and tissue specific characteristics of cis-regulatory elements are dependent on the specific promoter used and are best explored using endogenous promoter regions.^20, 21^ We have also found that the functional consequences of allelic variation only become significant following the activation of specific signalling pathways in specific cell types.^22^

On the basis of these previous studies we explored the hypothesis that SNPs in LD with rs9416742 contributed to susceptibility to MDD by altering the activity of highly conserved cis-regulatory elements in intron 3 of the *BICC1* gene on the cell specific and inducible activity of the *BICC1* promoter. We used a combination of LD studies, comparative genomics, *in situ* hybridisation, transgenic analysis and primary cell culture analysis to demonstrate the presence of a novel polymorphic cis-regulatory element in LD with rs9416742 that demonstrated significant allele specific effects on *BICC1* promoter activity in primary amygdala neurones. The significance of these finding in health and in the complex underpinnings of MDD are discussed.

## Materials and methods

### Bioinformatic analysis

Genomic analysis of the *BICC1* locus was performed using the UCSC genome browser (http://genome.ucsc.edu/). Allele frequency of SNPs was determined using the NCBI SNP database (http://www.ncbi.nlm.nih.gov/SNP/). Bioinformatic analysis of the effects of allelic variants on transcription factor binding sites were determined using the RegSNP website (http://viis.abdn.ac.uk/regsnp/Home.aspx).

### PCR amplification of regulatory regions, cloning- and site-directed mutagenesis

Because of its high GC content BICCprom was amplified using *BICC1*_Long_Prom_F and R oligonucleotide primers (Table 1) from human placental DNA using a GC-rich PCR system (Roche, East Sussex, UK). BICC77 was amplified using ECR rs2393477_F and R oligonucleotide primers (Table 1) from human placental DNA using the Expand High Fidelity PCR system (Roche). PCR products were cloned into pGEM-T-easy (Promega, Southamton, UK) and sequenced to confirm PCR fidelity and to determine genotype. To reproduce both haplotypes of BICC77 site-directed mutagenesis of BICC77 in pGEM-T Easy was undertaken using BICC1-SDM-61 primers (forward and reverse) and BICC1-SDM-77 (forward and reverse; Table 1) oligonucleotide primers using QuikChange II XL Site-Directed Mutagenesis Kit (Agilent, Edinburgh, Scotland, UK).

### DNA constructs

*pBICCprom-LacZ.* BICCprom was digested from pGEM-T easy using NcoI and EcoICRI and ligated into the NcoI and SmaI sites of the SB LacZ reporter plasmid (A kind gift from Sanbing Shen) to form pBICCprom-LacZ. *pBICCprom-Luc.* BICCprom was digested from pGEMT-easy using NcoI and SalI and ligated into the NcoI and XhoI sites of pGL4-23 (Promega). *pBICC77prom-Luc(CG and AA).* BICC77 (CG or AA) was digested out of pGEMT-easy using ZraI and SacI and placed in a ligation containing the pGL4.23 vector linearised with NcoI and Sac1 and BICCprom digested from pGEMT-easy using NcoI and EcoICRI.

### Transgenic analysis (including lacZ stain)

Following linearization and removal of the plasmid backbone, BICCprom-lacZ construct DNA was microinjected into one cell C57/BL6xCBA F1 mouse embryos at a concentration of 2–4 ng μl^−1^ as previously described.^23^ Surviving embryos were oviduct transferred into pseudopregnant CD1 host mothers. Transgenic mice from each line were humanely sacrificed by lethal injection of euthatol according to UK Home Office guidelines. These mice were then dissected to recover the brain which underwent submersion fixation in 4% PFA (paraformaldehyde disolved in phosphate buffered saline) for 4 h. All tissues were then stained using 5-bromo-4-chloro-3-indolyl-beta-D-galactopyranoside (X-gal) solution for 4–12 h as previously described.^23^ X-gal stained tissues were then prepared for vibratome sectioning by allowing tissue to equilibrate with increasing concentrations of sucrose (to 30%) in phosphate buffered saline at 4 °C. Tissues were equilibrated with bovine serum albumen/Gelatin solution (0.5 gelatin, 30 sucrose, 15.5% bovine serum albumen) overnight at 4 °C. Bovine serum albumen/gelatin embedded tissue was then hardened by addition of one-tenth of a volume of 25% gluteraldehyde. Fifty micron sections were then cut on a Vibratome series 1000 (Leica, Milton Keynes, UK). Sections were mounted on glass slides and photographed under light field or phase contrast illumination.

### *In situ* hybridisation

Radioactive *in situ* hybridisation was carried out as previously described.^24^ Mouse cDNA for use as a template for the production of antisense *BICC1* probe for radioactive *in situ* hybridisation was purchased from Source Bioscience (Genbank accession no. AK076154.1).

### Cell culture and luciferase reporter gene assays

Primary Amygdala cultures were prepared from P1-3 rat neonates as described earlier.^22^ Cells were transfected with plasmid DNA using Neuromag according to the manufacturer's instructions (OZ Bioscience, Marseilles, France). Briefly, Neuromag reagent was added to a plasmid-media preparation and incubated at room temperature for 15 min allowing 250 ng of plasmid DNA in the media per 150,000 cells to be transfected. The DNA-Media-Neuromag solution was then added to previously prepared single cell cultures in 24-well plates and incubated on a magnetic plate for 15 min in a cell culture incubator at 37 °C and 5% CO_2_. Luciferase activity of plasmid constructs were measured using dual luciferase assay kits (Promega) using lysates from cell cultures transfected with the GAL5.1 luciferase constructs. Cell cultures were lysed and the proteins stabilized using the passive lysis buffer as per the manufacturer's instructions (Promega). The dual-luciferase assay analysis was carried out on a Glomax 96-microplate luminometer (Promega) using 20 μl of cell lysate per well of a white 96-well plate.

### Data analysis

Sample sizes (*n*⩾3) were based on pooled amygdala neurones recovered from at least three individual rat neonates. Statistical significance of data sets were analysed using either two way analysis of variance (ANOVA) analysis with Bonferoni *post hoc* tests or using two tailed unpaired parametric Student *t*-test as indicated using GraphPad PRISM version 5.02 (GraphPad Software, La Jolla, CA, USA).

## Results

### *BICC1* displays spatially discrete mRNA expression within components of the limbic system

**Figure 1:**
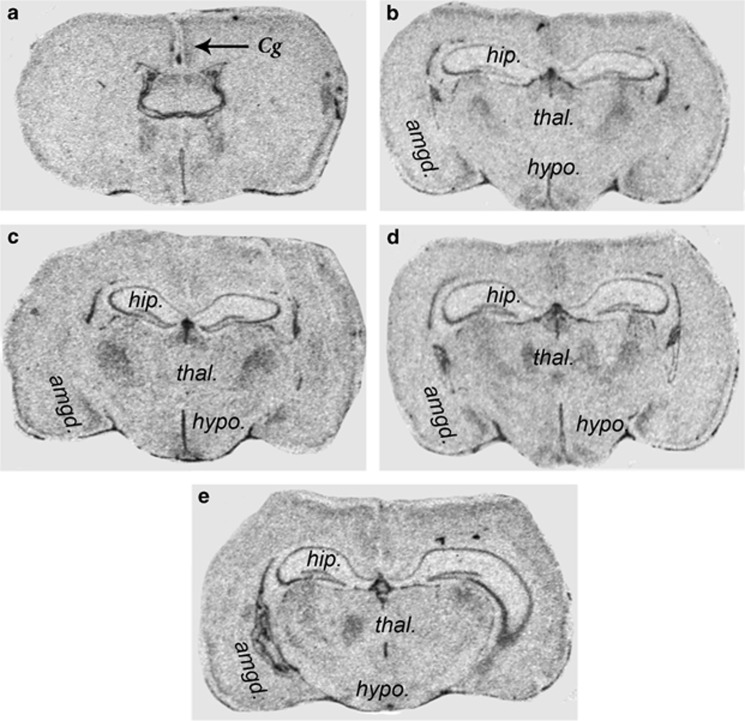
Autoradiographs of 10-μm coronal sections of adult male C57BL6 mouse midbrain (Bregma −0.34 to −2.70 mm) hybridised to a S^35^ labelled *BICC1* antisense RNA probe. amgd, amygdala; Cg, cingulate cortex; hip, hippocampus; hypo, hypothalamus; thal, thalamus.

Our previous GWA analysis provided a compelling case for examining the involvement of allelic variations within intron 3 of the *BICC1* gene in major depressive disorder.^7^ We also provided evidence that the long isoform of *BICC1* (isoform 1), that includes the first seven exons (Figure 3a), showed strong differential expression in specific nuclei of the human brain.^7^ In contrast, the short isoform (isoform 2, lacks the first 7 and last 3 exons), although detectible in kidney and bone tissue, demonstrated consistently low expression in all brain tissues.^7^ These observations argue against a role for the short isoform in brain function and, for this reason, we concentrated our analysis on the long isoform. Although our microarray analyses were highly informative they lacked the resolution to determine the specific regions of the brain that expressed *BICC1*. For this reason we used *in situ* hybridisation using probes derived from the full length *BICC1* complementary DNA that reflects the long isoform, on sections of adult male mouse brain to determine whether *BICC1* mRNA was expressed throughout the brain or displayed discrete regions of expression. These *in situ* analyses demonstrated that, although full length *BICC1* transcript expression was present in the majority of tissues at low levels, areas of higher expression were evident within discrete components of the limbic system including the hippocampus, the hypothalamus and the amygdala (Figure 1). There was also evidence of increased expression within the cingulate cortex and the thalamus (Figure 1). Because the limbic system plays a critical role in conditioned fear and mood modulation these observations support a role for changes in the expression of *BICC1* long isoform in contributing to mood disorders.

### Bioinformatic analysis of the *BICC1* promoter

We have established that *BICC1* mRNA demonstrates tissue specific expression patterns within regions of the mouse limbic system. These findings are consistent with evidence of a role for SNP variants within intron 3 of the *BICC1* gene in MDD. Because these SNPs are non-coding we next explored the hypothesis that they influenced mood by altering the transcriptional regulation of *BICC1* within the limbic system. However, we have no knowledge of the systems regulating *BICC1* mRNA expression within the brain. For this reason we used information contained within the UCSC genome browser to direct our studies and provide evidence of the promoter regions supporting *BICC1* expression in the brain. UCSC describes the existence of two mRNA species transcribed and spliced from the human *BICC1* gene locus (Supplementary Figure 1A and Figure 3a). Thus, the short splice form is encoded by 10 exons distributed over 21 kb (GRCh37/hg19, chr10:60,553,182-60,574,087) and the long splice form shares the same 10 exons but has an additional 11 exons distributed over 318 kb (GRCh37/hg19, chr10:60,272,942-60,591,445) (see Supplementary data Supplementary Figure 1a.). Previous mRNA expression analysis of the short and long splice-forms of *BICC1* suggested that, although expression of both splice-forms was detected in specific human neuronal tissues and cells, the longer splice-form was expressed at considerably higher levels and varied strongly between different tissues suggesting that its expression is maintained by a stronger promoter element.^7^ To confirm this observation we used data embedded in the ENCODE data set to explore the promoter characteristic of DNA sequence around the transcriptional start sites of the long and short splice-forms. Using accepted criteria such as histone modification patterns (H3K4me2, H3K4me3, H3K9ac and H3K27ac) as well as FAIRE and DNAse1 hypersensitivity patterns characteristic of promoter regions in a number of different human cell lines (GM2878, HeLa, HepG2 and embryonic stem cells etc.) we recovered data consistent with higher levels of promoter activity around the transcriptional start site of the long splice-form in comparison to the short isoform which demonstrated fewer active promoter characteristics in all cell lines used (Supplementary Figure 1b–e and Figure 3a) consistent with its lower activity in microarray analysis reported by Lewis *et al.*^7^ Furthermore, the promoter of the long splice-form, was more highly conserved outside of the coding region than the promoter region of the short splice form (Supplementary Figure 2b and e). Because of this evidence we focussed our subsequent efforts on the promoter of the long splice form.

### Cloning and characterisation of the *BICC1* promoter

**Figure 2:**
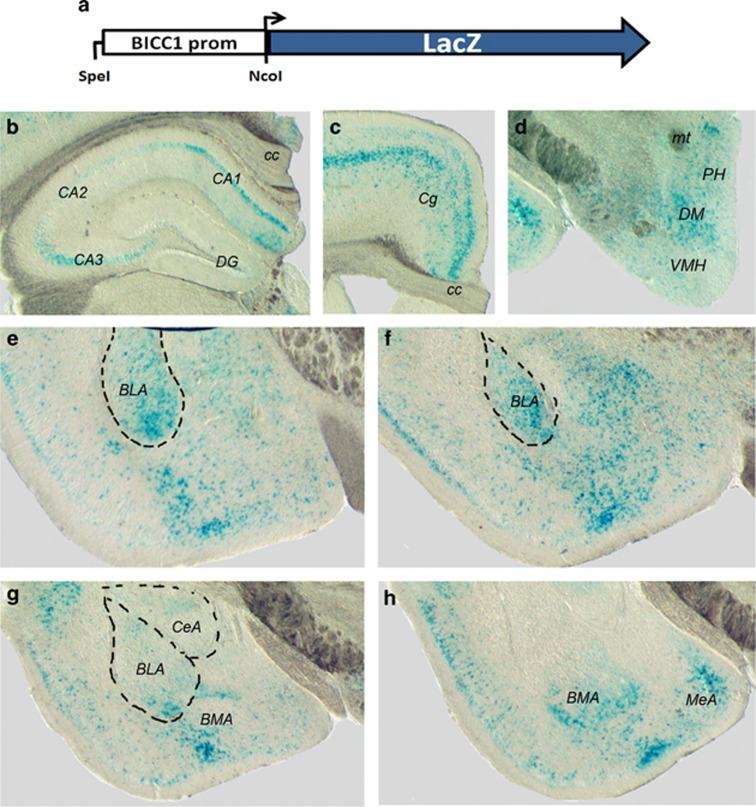
(**a**). Diagrammatic representation of the BICC1prom-LacZ reporter constructs (not to scale). (**b-h**) photomicrographs of coronal sections of mouse midbrain transgenic for the BICC1prom-LacZ plasmid and stained with X-Gal to detect the expression of LacZ from the transgene in the hippocampus (**b**), the cingulate cortex (**c**), the hypothalamus (**d**) and the amygdala (**e-h**). BLA, basolateral amygdaloid nucleus; BMA; basomedial amydaloid nucleus; LacZ, β-galactosidase gene; CA1-CA3, CA regions of hippocampuscc, corpus colosum; CeA; central amygdaloid nucleus; Cg, cingulate cortex; DG, dentate gyrus; DM, dorsomedial hypothalamic nucleus; MeA, medial amygdaloid nucleus; mt, mammillothalamic tract; VMH, ventromedial hypothalamic nucleus; PH, posterior hypothalamic area; VMH, ventromedial hypothalamic nucleus.

To isolate and characterise the long isoform promoter (henceforth referred to as BICC1prom) we designed PCR primers (see Table 1) to isolate a 691 bp region of the *BICC1* locus that spanned both the GC enriched region (75% GC content) and the region most conserved in vertebrates (Supplementary Figure 2a). This sequence also contained the first exon of the long isoform of *BICC1*. Following amplification BICC1prom was cloned into a LacZ reporter plasmid using an NcoI site that coincided with the transcriptional start site of both *BICC1* and the reporter *LacZ* gene (Figure 2a). Using pronuclear microinjection and oviduct transfer this reporter construct was used to generate a number of transgenic mouse lines (*n*=6). Brain tissues were recovered from these lines and subjected to X-Gal staining. Two of the transgenic mouse lines demonstrated robust and consistent staining for the expression of the *LacZ* gene in areas of the cingulate cortex, hippocampus, thalamus and in specific nuclei of the amygdala including the basolateral, basomedial, central and lateral amygdaloid nuclei (Figures 2a–h). Because both lines produced similar expression patterns in these areas it is highly unlikely that the patterns of expression generated represented insertional effects but were representative of BICC1prom activity. The activity of the human BICC1prom within these components of the limbic systems was entirely consistent with its role in supporting the expression of mRNA that we describe above.

### The *BICC1* promoter is active in primary amygdala neurones

**Figure 3:**
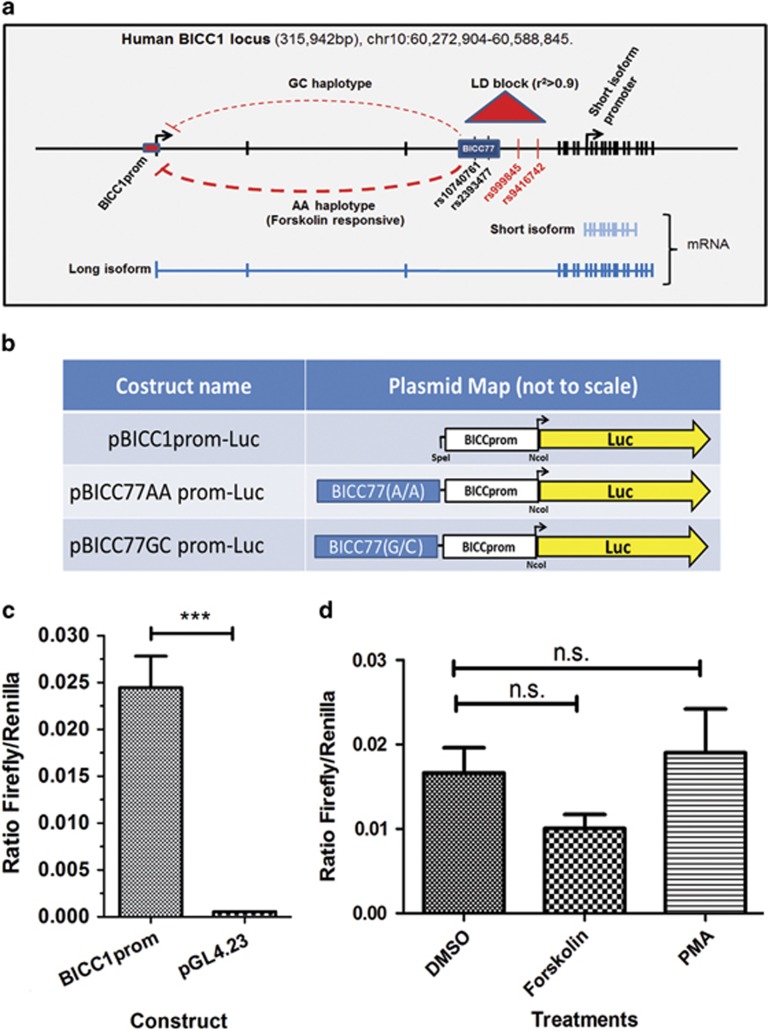
(**a**) A diagrammatic representation (not to scale) of the *BICC1* locus demonstrating the linear relationships between the genomic locus (black line) and the short (light blue lines) and long (dark blue lines) mRNA isoforms. Exons are indicated by heavy vertical lines. Long and short isoform promoters are indicated using bent black arrows. The *BICC1* promoter and BICC77 are represented by a red filled box and a blue filled box respectively and represents a distance of 260 kb. Sites of MDD associated SNPs are represented by thin red vertical lines and conserved SNPs in strong linkage disequilibrium are indicated by thin black vertical lines. The region of the *BICC1* locus in strong LD is represented by a red triangle. The functional relationships suggested in the current manuscript are summarised using broken red lines to indicate the increased repressive effects of BICC77AA on BICC1prom compared with BICC77GC in primary amygdala neurones. (**b**) diagrammatic representation of the luciferase reporter constructs used in subsequent experiments. (**c**) A bar graph representing the results of a dual-luciferase analysis comparing the activity of the pBICCprom-Luc vector (BICCprom) to that of pGL4.23 following magnetotransfection into primary amygdala neurones. (**d**) Effects of treatment of primary amygdala neurones magnetofected with the pBICCprom-Luc plasmid and treated with 10 μM forskolin or 10 μM PMA for 16 h before dual-luciferase analysis. *n*=15. MDD, major depressive disorder; N.S., not significant; PMA, phorbol 12-myristate 13-acetate; SNP, single-nucleotide polymorphism. Error bars are standard error of the mean, *n*=4, analysis using (**c**) two tailed unpaired *t*-test or (**d**) ANOVA. n.s. not significant, ***P<0.005.

The analysis of BICC1prom in transgenic lines using a LacZ reporter construct reflected the cell specific distribution of *BICC1* mRNA in limbic regions of the brain (Figure 2). However, the analysis of LacZ constructs using transgenic mice does not represent an effective method of assaying the quantitative effects of other regulatory regions, SNPs or activation of signal transduction pathways on BICC1prom. For this reason we cloned BICC1prom into the NcoI site of a firefly luciferase plasmid (pGL4.23; Promega) replacing the generic TATA box promoter to form pBICC1prom-Luc (Figure 3b). Because of the role of the amygdala in mood and fear behaviour we magnetofected BICCprom-luc into cultured primary rat amygdala neurones together with the renilla luciferase vector pGL4.74 and in parallel with the pGL4.23 backbone plasmid. This demonstrated that the *BICC1* promoter was over 40 times more active in amygdala derived neurones that the minimal TATA box promoter of the pGL4.23 plasmid that was used as the basis of our luciferase promoter constructs (Figure 3b). These observations further support a role for the *BICC1* promoter in the amygdala.

### BICC1prom is moderately repressed by PKA activation but not PKC activation

It is well established that modulation of the activity of protein kinase A (PKA) pathways in the amygdala play a critical role in the consolidation of fear memories.^25, 26^ There is also evidence that components of the protein kinase C (PKC) pathway are activated in the amygdala during fear conditioning^27^ where they may also have a role in fear learning.^28, 29^ Due to the known involvement of PKA and PKC pathways in fear memory and learning we explored the effects of activation of these pathways on the activity of the BICC1 promoter in primary amygdala neurones. We found little evidence that the PKC pathway could influence BICC1prom activity in primary amygdala neurones (Figure 3c). We found some evidence of negative regulation of BICCprom by activation of the PKA pathway using forskolin but this was not statistically significant (Figure 3c).

### Linkage disequilibrium analysis of rs9416742 and rs999845

We have provided evidence that BICC1prom is responsible for supporting the discrete expression of *BICC1* mRNA in the limbic system. These studies provide a useful platform for further exploring the possible effects of mood disorder associated allelic variants in *BICC1* intron 3 on the tissue specific activity of BICC1prom. Using genome wide association studies we previously demonstrated that a significant association existed between the rs9416742 and rs999845 polymorphisms (*P*=1.3 × 10^−7^ and 3.14 × 10^−7^, respectively) and susceptibility to MDD.^7^ As previously mentioned neither of these SNPs occur within any coding regions of the *BICC1* gene nor were they in LD with coding SNPs. Because evolutionary conservation has been shown to be highly suggestive of functionality we explored the hypothesis that rs9416742 and rs999845 occurred within conserved cis-regulatory regions. Unfortunately, comparative genomics demonstrated that neither SNP occurred within a region of the *BICC1* locus which was highly conserved or displayed markers indicative of cis-regulatory regions. However, both SNPs were found to be in strong linkage disequilibrium with a SNP (rs2393477) that occurred in a 200 base pair region of human BICC1 intron 3 that had been highly conserved in mammalian species as divergent from humans as marsupials (opossum) and monotremes (platypus) (Figure 3a and Supplementary Figure 3). This high degree of deep conservation (‘depth' refers to evolutionary time as opposed to degrees of conservation by pairwise comparison) strongly suggests that this sequence has an important biological function and has served in maintaining species fitness during the 210 million years since placental mammals and monotremes diverged.^30^ This region of homology was also 260 kb away from BICC1prom which is well within the known range of influence of many cis-regulatory elements. Other regions of *BICC1* intron 3 between this cluster of SNPs were also found to be highly conserved and also contained polymorphisms but these were not found to be in significant LD with either rs9416742 or rs999845 (Figure 3a and Supplementary Figure 3).

### BICC77 represses the activity of BICC1prom in amygdala neurones in an allele specific manner

**Figure 4:**
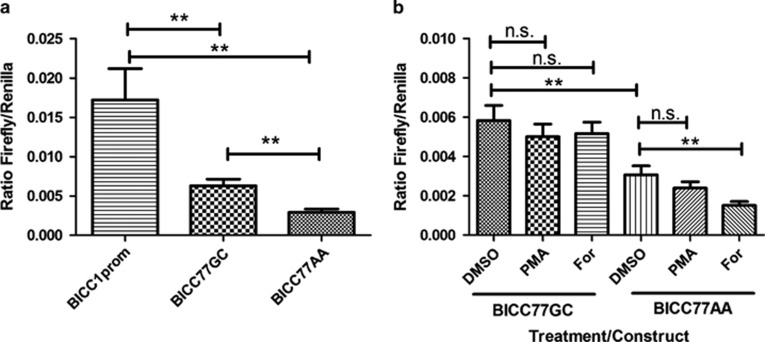
(**a**) Bar graphs showing the results of dual-luciferase analysis on primary amygdala neurones magnetofected with pBICC1prom-Luc (BICC1prom), pBICC77GCprom-Luc (BICC77GC) or pBICC77AAprom-Luc (BICC77AA) (*n*=9). (**b**) The results of dual-luciferase analysis of primary amygdala neurones magnetofected with either the pBICC77GCprom-Luc (BICC77GC) or pBICC77AAprom-Luc (BICC77AA) and treated with DMSO, forskolin (For) or PMA. All plasmids were co-transfected with the pGL4.74 renilla luciferase plasmid (*n*=10). **P<0.01. Error bars represent the standard error of the mean. All analysis in Figure 4 undertaken using two way ANOVA with Bonferroni *post hoc* tests. ANOVA, analysis of variance; DMSO, dimethyl sulfoxide; N.S., not significant; PMA, phorbol 12-myristate 13-acetate.

To determine whether allelic variants of highly conserved regions of *BICC1* intron 3 affected the activity of BICC1prom in amygdala neurones we designed PCR primers (BICC77 for. and BICC77 rev.; Table 1) to isolate a highly conserved 200 bp region of *BICC1* intron 3 (BICC77) that contained rs2393477 and its close neighbour rs10740761, which was in high LD (*r*^2^>0.95). BICC77 was then subjected to two rounds of site-directed mutagenesis (SDM; see Table 1 for SDM primers used) to recreate allelic variants of rs2393477 and its close neighbour rs10740761 and sequenced to confirm SDM fidelity. Different BICC77 haplotypes were cloned into BICCprom-luc to form pBICC77AA-Luc and pBICC77CG-Luc (Figure 3a). Magnetofection of these constructs into primary amygdala cells demonstrated that BICC77 strongly repressed *BICC1* promoter activity in these cells. Interestingly, BICC77AA had a significantly stronger repressive effect that did BICC77GC (Figures 3a and 4a).

### BICC177 further represses BICC1prom activity following PKA but not PKC activation.

We have shown that allelic variants of the highly conserved sequence BICC77 are able to differentially down regulate the activity of BICC1prom within primary amygdala neurones. Because of the essential role played by cis-regulatory elements in relaying signal transduction information to promoter regions^22^ we next explored the possibility that allelic variants of BICC77 differed in their ability to relay signal transduction signals to BICC1prom. We treated primary amygdala cultures transfected with allelic variants of pBICC77prom-Luc (AA and CG) with forskolin or PMA. PMA had no significant effect on the activity of either allelic variant and forskolin did not significantly alter the ability of BICC77GC to repress BICC1prom activity (Figure 4b). However, BICC77AA demonstrated significantly higher repression of BICC1prom activity following treatment with the PKA agonist forskolin (Figures 3a and 4b).

### Bionformatic analysis of transcription factor binding at rs2393477 and rs10740761.

We asked whether it was possible to use a bioinformatics approach to identify candidate transcription factors whose binding to BICC77 was allele dependent. We used the RegSNP website to determine whether the AA haplotype altered the predicted binding efficiency of known transcription factors compared with the protective GC haplotype. Examination of rs10740761 using RegSNP shows that the C allele potentially binds the transcription factor Deaf1 which is suspected in causing memory deficits and anxiety like behaviour if deleted from the mouse genome.^31^ The C allele of rs10740761 was predicted to bind Deaf 1 with a matrix score of 0.7323 wheras the A allele only had a matrix of 0.5. The A allele of rs2393477 was predicted to bind GATA factors (1, 2, 3 and 6) with much higher affinity (matrix score for C allele=0.7 and for T allele 0.92). This is intriguing as overexpression of GATA factors has been associated with depressive behaviour^32^ and a highly conserved C/EBP binding site exists just next to this locus. This arrangement of transcription factor binding has been associated with PKA activation on the past.^33^

## Discussion

To locate the presence of a human genomic locus that could contribute to susceptibility to major depressive disorder we carried out a GWA analysis of a large patient cohort and succeeded in identifying two SNPs within intron 3 of the *BICC1* gene with significant association. We observed that the T allele of the most strongly associated SNP; rs9416742, that has a frequency of 0.235 in healthy individuals, was found to have a protective affect against depression. Although neither of these SNPs were found within the coding region of *BICC1*, or within an area of intron 3 that could be identified as a cis-regulatory region, they were found to be in strong linkage disequilibrium with a SNP within a region of intron 3 that had been conserved for 210 million years thus representing the majority of the extent of mammalian evolution. We isolated this conserved region, that we called BICC77, and reproduced both of the allelic forms present in the human population (referred to as AA and GC). Because these SNPs lay within the *BICC1* gene and because subsequent analysis had demonstrated that the allelic variants of *BICC1* in question were associated with greater hippocampal volumes^13^ we tested the hypothesis that allelic variants of BICC77 differentially regulating the transcription of the *BICC1* gene in the brain.

To test this hypothesis it was necessary to first determine whether *BICC1* was expressed in any areas of the brain known to control mood. We used *in situ* hybridisation to demonstrate that *BICC1* mRNA was more strongly expressed in specific areas of the limbic system including the hippocampus, amygdala and hypothalamus. Subsequent analysis of existing genomic databases succeeded in identifying the active *BICC1* promoter by virtue of its extent of conservation, its accessibility to FAIRE and DNAseI analysis and its interaction with known promoter histone marks. Using magnetofection of primary amygdala cells with luciferase markers and dual-luciferase assays we were able to show that this promoter region was highly active in primary amygdala neurones compared with a generic TATA box promoter. Furthermore, by generating two different lines of transgenic mice with the *BICC1* promoter linked to the *LacZ* gene we also demonstrated pronounced tissue specificity in the activity of the *BICC1* promoter in hippocampus, amygdala and hypothalamus. These analyses established the expression of *BICC1* and the activity of its promoter in regions of the limbic system that have critical roles roles in mood modulation and depressive behaviour. In addition, identifying the tissue specific activity of the *BICC1* promoter provided us with a useful platform for studying the effects of allelic variants of BICC77 on its activity and hence the activity of the *BICC1* gene.

We next asked what effect BICC77 would have on the activity of the *BICC1* promoter in primary amygdala neurones and we were able to demonstrate that BICC77 acted as a strong repressor of *BICC1* promoter activity within these cells. We have previously observed this model in the regulation of *BDNF* promoter 4 whereby a highly conserved region of the *BDNF* locus, containing a SNP associated with depression (BE5.2), caused significant repression of *BDNF* promoter 4 within 3 different primary cell types (hippocampus, cortex and amygdala).^22^ These analyses of the *BDNF* locus suggested an important role for selective negative regulation in the modulation of inducible gene expression in the brain. In combination with this previous study, the current study highlights the complexities of understanding the specific mechanisms by which depression associated allelic variants may alter pathological susceptibility. Thus, by subtly changing the delicate homoeostatic balance of negative and positive regulatory mechanisms required to maintain levels of gene expression essential for mental health differences in the activity or regulatory elements over a life time, or following trauma, may be sufficient to contribute to mental illness. There is every reason to believe that identifying the effects of allelic variation on negative regulation will be critical for understanding the causes of psychiatric pathologies including depression.

With this concept in mind we then asked whether allelic variants of BICC77 differed in their ability to negatively regulate the activity of the *BICC1* promoter in amygdala cells. We recreated the different genotypes of BICC77 present in the human population (AA and CG) and found that the minor haplotype of BICC77(GC), that is in LD with the protective T allele of rs9416742, was not as potent a repressor as the major allele (AA). Whilst there is currently no evidence to suggest that levels of *BICC1* expression within individuals with the CG haplotype of BICC77 are higher than those with the AA haplotype, the observation of a significant difference in the influence of these haplotypes on the activity of the *BICC1* promoter in primary amygdala neurones provides us with a possible insight into a mechanism contributing to mood disorders. These observations reflect the results of a previous study, which demonstrated that the CA haplotype of the GAL5.1 enhancer, that supports expression of the galanin neuropeptide in areas of the brain including the paraventricular nucleus and amygdala, was significantly weaker than the GG haplotype in primary hypothalamic neurones.^19^ Subsequent analysis of these haplotypes in human cohorts suggested that the CA haplotype was protective against alcohol and cannabis abuse in certain populations^34, 35^

It is accepted that one of the main roles of gene regulatory regions is to interpret signal transduction information within the surrounding cell cytoplasm for the transcriptional machinery (RNA polymerase TFIID and mediator) bound at the promoter region of genes. In the specific case of the amygdala, a number of signal transduction pathways have been implicated in modulating the cellular systems that control mood.^36^ One of the best studied is the PKA signal transduction pathway that is activated in amygdala neurones by binding of stress released epinephrine to B1 adrenergic receptors.^36^ Because of the known contribution of chronic stress in the exacerbation of mood disorders we sought to determine the effects of agonists of signal transduction pathways such as PKA and PKC known to have a role in amygdala function. Treatment of transfected cultures to activate the PKC pathway had no effect on BICC77 and no allelic differences were observed. Activation of PKA pathways in transfected cells also had little or no effect on the ability of the BICC77GC haplotype to repress the *BICC1* promoter. Intriguingly, however, forskolin treatment resulted in further repression of the *BICC1* promoter by BICC77AA. Further bioinformatic analysis suggested that the AA haplotype had an increased affinity with a combination of transcription factors (GATA and C/EBP) that have previously been associated with PKA response.^33^ Because the AA haplotype of BICC77 is the major haplotype within the human population and also represents the ancestral haplotype present in all other mammals, it can be hypothesised that the CG allele of BICC77, which is in LD with the protective T allele of rs9416742, is not only less repressive of *BICC1* promoter activity but has also lost its ability to respond to activation by PKA modulated signalling inputs. Indeed, it could be hypothesised that the reduction in its ability to repress the BICC1 promoter or to respond to PKA signalling may be mechanistically related to the protective effects observed for the T allele of rs9416742.

## Conclusion

Major depressive disorder has been shown to be a highly complex disease state to diagnose and this may reflect the large numbers of unreplicated genome wide association studies hits associated with this condition (>100; HuGE genome navigator). There is also increasing evidence of the possibility of masking or exacerbation of depression phenotypes through epigenetic influences that are altered following early life traumatic events that may have a role in reducing the efficacy of genetic association analysis.^37, 38^ However, the overwhelming conclusion that has been drawn from the results of these studies is that the genetic and epigenetic influences in susceptibility to mood disorders are unlikely to be solely due to changes in gene coding regions but will instead affect the mechanisms that control the spatial, tissue specific and inducibility of these genes. The current manuscript describes the use of novel combinations of different techniques to determine the mechanisms through which alleles associated with MDD might contribute to susceptibility. Thus, in addition to suggesting a novel contributory mechanism towards MDD susceptibility the current manuscript describes innovative approaches to exploring the mechanisms through which non-coding disease associated SNPs may contribute to disease.

## Figures and Tables

**Table 1:** Names and sequences of oligonucleotides used in the construction of the plasmid reporter constructs described in the manuscript

*Oligonucleotide*	*Sequence (5'-3')*
BICC1_Long_Prom_F	GTGGCAGTGTGTACGTGGTG
BICC1_Long_Prom_R	AAAAGGAGTTAGAGGCTCAGTCG
ECR rs2393477_F	TTCTTAGCCTTTCTGAAAATCAGTAG
ECR rs2393477_R	TTAACTCTGTTTTCTGCCAGAGTGAG
BICC1-SDM-61 F	GGAATATTATTTTTAACCTATCTAACGTATGGAGTTGACCCCCTAAAAG
BICC1-SDM-61 R	CTTTTAGGGGGTCAACTCCATACGTTAGATAGGTTAAAAATAATATTCC
BICC1-SDM-77 F	CATATTTCTCAAAGGTCCTATAAGCATTACAACACATTTATCAAAG
BICC1-SDM-77 R	CTTTGATAAATGTGTTGTAATGCTTATAGGACCTTTGAGAAATATG

Abbreviation: BICC1, bicaudal C homolog 1.
