# Supplementary figures and images for: Analysis of the effects of depression associated polymorphisms on the activity of the BICC1 promoter in amygdala neurones
Source: Pharmacogenomics J. 2015 Oct 6;16(4):366–74. doi: 10.1038/tpj.2015.62 (PMC4973013; doi:10.1038/tpj.2015.62)

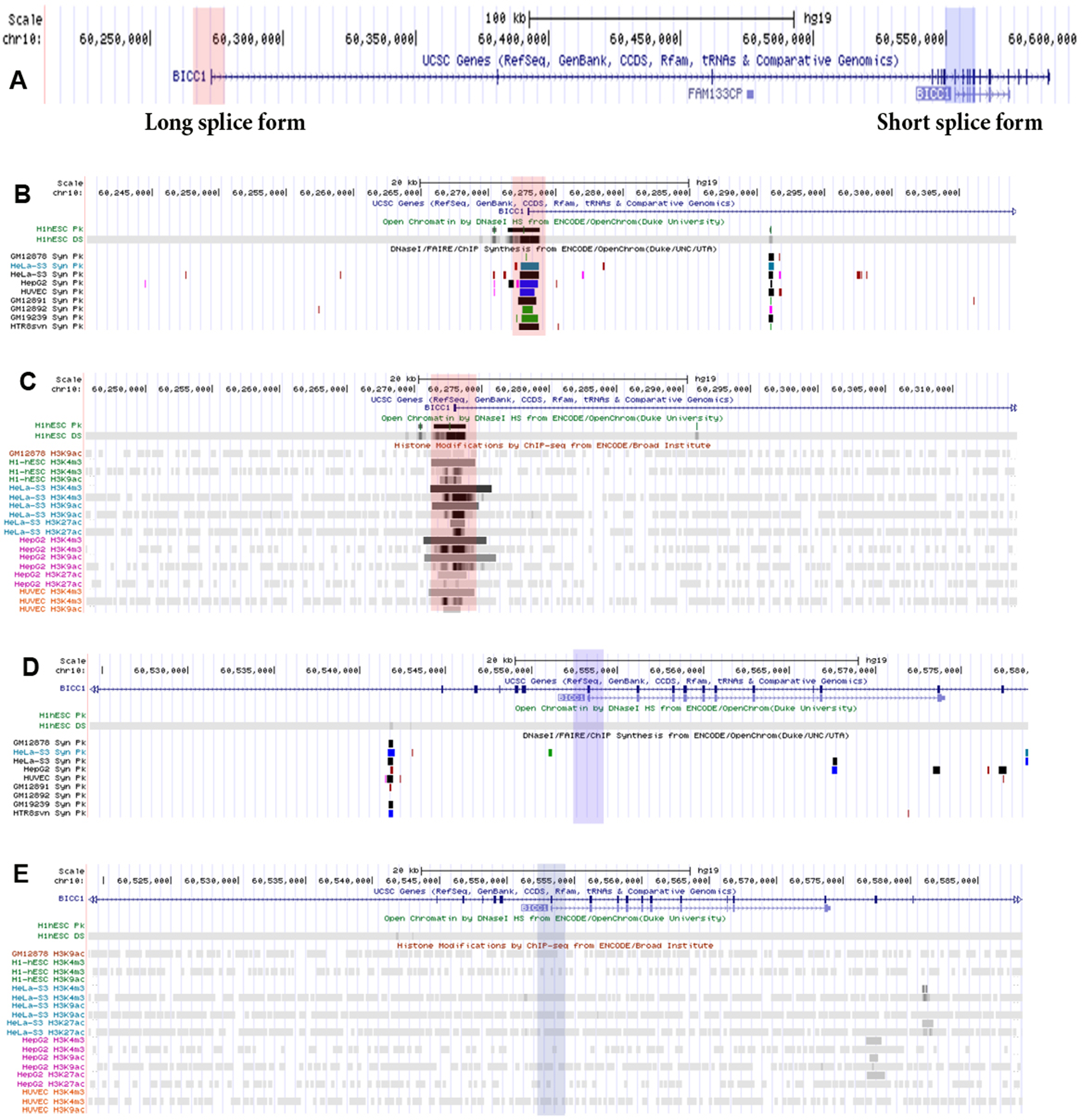

Supplement: Supplementary Figure 1 [file tpj201562x1.tif]

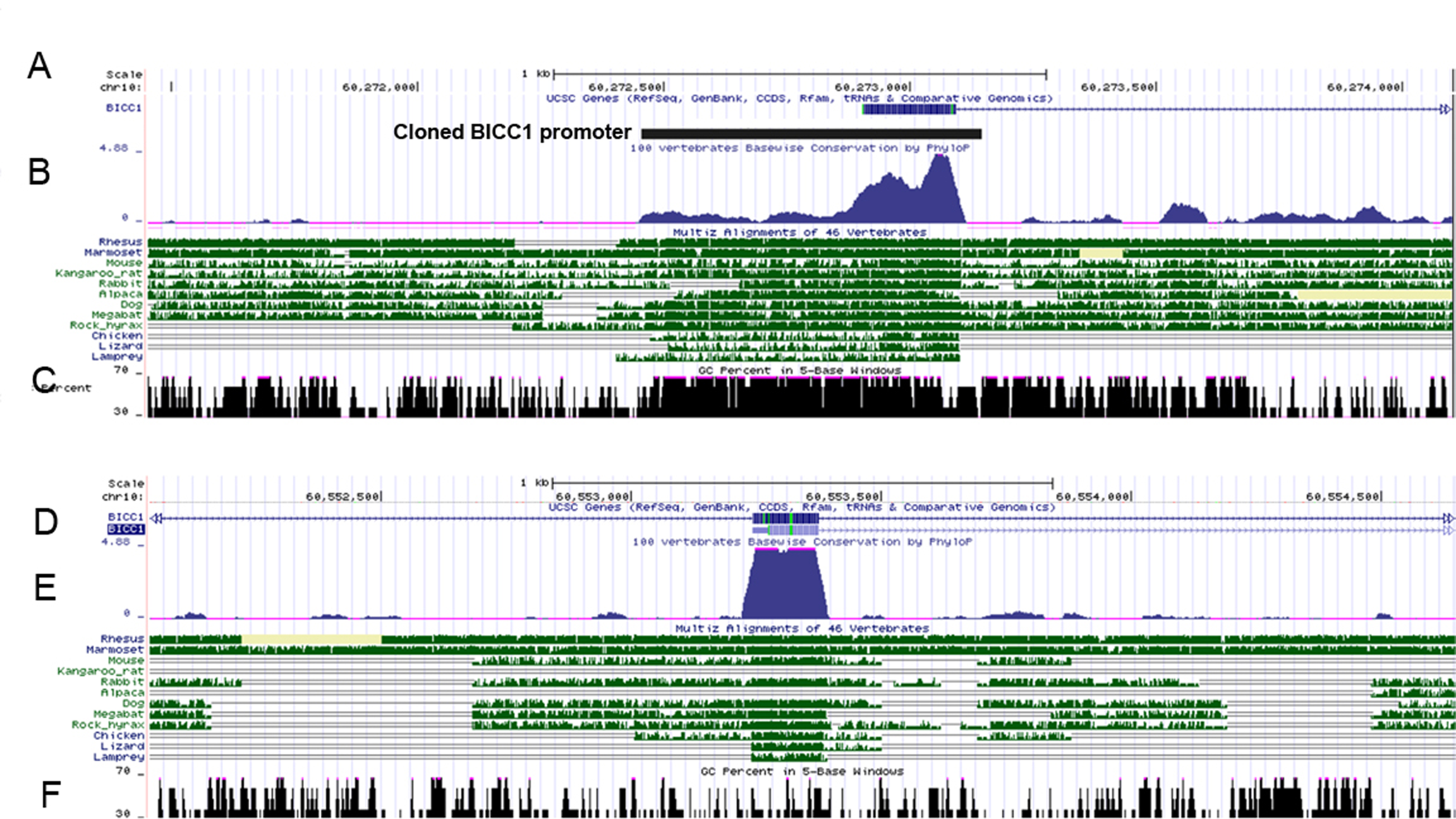

Supplement: Supplementary Figure 2 [file tpj201562x2.tif]

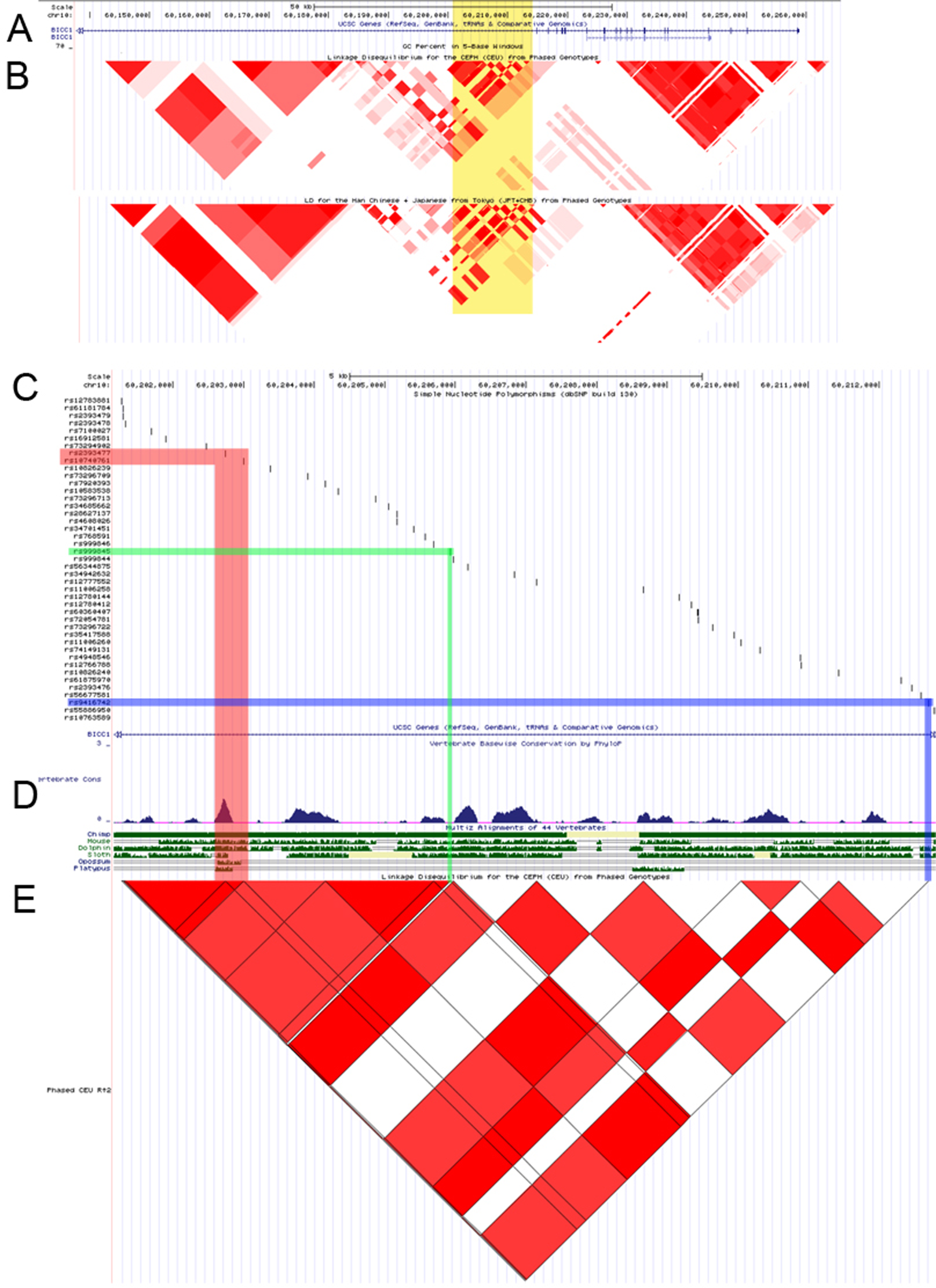

Supplement: Supplementary Figure 3 [file tpj201562x3.tif]
