# Supplementary material for: Analysis of the effects of depression associated polymorphisms on the activity of the BICC1 promoter in amygdala neurones
Source: Pharmacogenomics J. 2015 Oct 6;16(4):366–74. doi: 10.1038/tpj.2015.62 (PMC4973013; doi:10.1038/tpj.2015.62)
Supplement: Supplementary Figure Legends [file tpj201562x4.doc]

**Supplementary figure 1.** ENCODE genomic data derived from the UCSC genome browser (<https://genome.ucsc.edu/index.html>) comparing the activity (DNase1 and FAIRE analysis) and histone binding activity (ChIP-seq) of the promoters of the “long” and “short” (light blue filled box) splice forms of the BICC1 gene. **A.** linear representation of the “long” (Dark blue lines) and “short” (light blue lines) mRNA splice forms of the BICC1 coding region showing scale bar (100kb) and co-ordinates. The pink filled box denotes the area of the promoter of the long isoform described in **B** and **C** and blue filled box denotes areas of the promoter of the short isoform described in **D** and **E**. **B** and **D** Tracks from ENCODE at Duke University and the Broad Institute showing DNase1 and FAIRE analysis of **(B**) the “long” splice form promoter and (**D)** the “short” splice form promoter in different cell types (See UCSC browser for more detailed key). Green/black/blue bands represent areas that are de-condensed and transcriptionally active. **C** and **E** Tracks from ENCODE at Duke University and the Broad Institute showing ChIP–seq data using antibodies against different methylation and acetylation states of the histone 3 (H3) protein interacting with **(C)** the “long” splice form promoter and **(E)** the “short” splice form promoter in a number of different tier 1 and 2 cell lines (See UCSC browser for Key). Black bands/smears indicate regions where H3 with indicated modifications bind the BICC1 locus.

**Supplementary figure 2.** ENCODE genomic data from the UCSC genome browser demonstrating **(A)** the region of DNA cloned to represent the BICC1 promoter. **B** and **E**  comparing levels of sequence homology in 100 vertebrates over the promoters of the “long” (B) and **(E)** “short” splice forms (Blue peaks and green bands). **C** and **F** graph demonstrating densities of CpG dinucleotides in each promoter.

**Supplementary Figure 3.** Encode Genomic data derived from the UCSC genome browser showing (A) the extent of the coding region of BICC1. **B** Inverted mountain plots demonstrating degrees of linkage dis-equilibrium (LD, r^2^) between SNPs within the BICC1 locus within European (CEU) and East Asian (JPT and CHD) populations­­­­. Increased intensity of red denotes increasing levels of LD (r^2^= 0.75-1.0) and white denotes random segregation of alleles. The yellow filled box denotes the area of BICC1 intron 3 denoted in **C-E.** **C**, a list of relevant SNPs (not exhaustive) found within intron 3 and their location with respect to **D** and **E**. Filled boxes highlight the positions of SNPs (green, rs999845 and blue, rs9416742 ) associated with MDD in Lewis et al 2010. Red filled boxes indicate the extent of the BICC77 region cloned and the SNP making up the different haplotypes. **D**, a demonstration of levels of conservation of sequences within BICC1 intron 3 and **(E)** inverted mountain plots demonstrating levels of LD existing between these SNPs in European populations (CEU).
